# The awareness of chronic kidney disease and aging; the focus of world kidney day in 2014

**Published:** 2014-01-01

**Authors:** Hamid Nasri

**Affiliations:** Department of Nephrology, Division of Nephropathology, Isfahan Kidney Diseases Research Center, Isfahan University of Medical Sciences, Isfahan, Iran

**Keywords:** World kidney day, Diabetic nephropathy, Renal failure

**Implication for health policy/practice/research/medical education:**

The global population is aging, and the number of persons over the age of 85 years is increasing faster than any other age group. As the population continues to age, physicians encounter expanding numbers of older individuals with chronic renal failure which is a global contest as a non-communicable epidemic. World kidney day has a purpose for everyone to care for his kidneys and check if, they are at risk for kidney disease. Additionally prevention and early detection are the critical points of the aim in world kidney day.

The incidence of chronic kidney disease is high in older persons and appears to be increasing (1,2), in contrast, many studies have shown that, chronic kidney disease is a common condition that stimulates of premature aging, cellular senescence and through toxic alterations in the internal milieu (3-6). The main question however bear in mind is the importance of chronic kidney disease in elderly patients. Indeed age is a major effect modifier amongst patients with chronic kidney disease (7-9). Among patients of all ages, rates of both death and end-stage kidney failure were inversely related to glomerular filtration rate at baseline. However, amongst those with comparable levels of glomerular filtration rate, older patients had higher rates of death and lower rates of end-stage kidney failure than younger patients (2-8). Older population are particularly susceptible to renal injury from age-related decline in glomerular filtration as well as renal injury from various chronic disease states for instance, hypertension, diabetes mellitus, glomerular, or tubulointerstitial disorders. Despite the fact that glomerular filtration rate gradually declines with age, whether this declining of renal function is part of a normal ageing process is in doubt (6-8). Various evidence has recently been shown that, the presence of chronic kidney disease is an independent contributor to risk factor for weakening in physical and cognitive functions in older adults too (6-9). Chronic renal insufficiency affects 45% of persons older than 70 years of age and can twofold the risk for physical diminishing, cognitive dysfunction, and frailty (9-12). In this regard, world kidney day has a purpose for everyone to care for his kidneys and check if, they are at risk for kidney disease. Additionally prevention and early detection are the critical points of the aim in world kidney day (9-12).

## Author’s contribution

HN is the single author of the manuscript.

## Conflict of interests

The author declared no competing interests.

## Ethical considerations

Ethical issues (including plagiarism, misconduct, data fabrication, falsification, double publication or submission, redundancy) have been completely observed by the author.

## Funding/Support

None declared.
